# Environmental tobacco smoke increases autophagic effects but decreases longevity associated with Sirt-1 protein expression in young C57BL mice hearts

**DOI:** 10.18632/oncotarget.9176

**Published:** 2016-05-04

**Authors:** Wei-Jen Ting, Jaw-Ji Yang, Chia-Hua Kuo, Zi-Jun Xiao, Xin-Ze Lu, Yu-Lan Yeh, Cecilia-Hsuan Day, Su-Ying Wen, Vijaya Padma Viswanadha, Chong-He Jiang, Wei-Wen Kuo, Chih-Yang Huang

**Affiliations:** ^1^ The Sixth Affiliated Hospital of Guangzhou Medical University, Guangdong, China; ^2^ Graduate Institute of Basic Medical Science, China Medical University, Taichung, Taiwan; ^3^ Institute of Medicine, School of Dentistry, Chung-Shan Medical University, Taichung, Taiwan; ^4^ Department of Sports Sciences, University of Taipei, Taipei, Taiwan; ^5^ National Taichung First Senior High School, Taichung, Taiwan; ^6^ Department of Pathology, Changhua Christian Hospital, Changhua, Taiwan; ^7^ Jen-Teh Junior College of Medicine, Nursing and Management, Miaoli, Taiwan; ^8^ Department of Nursing, Mei Ho University, Pingtung, Taiwan; ^9^ Graduate Institute of Clinical Medical Science, China Medical University, Taichung, Taiwan; ^10^ Department of Dermatology, Taipei City Hospital, Renai Branch, Taipei, Taiwan; ^11^ Department of Biotechnology, Bharathiar University, Coimbatore, India; ^12^ Department of Biological Science and Technology, China Medical University, Taichung, Taiwan; ^13^ Graduate Institute of Chinese Medical Science, China Medical University, Taichung, Taiwan; ^14^ Department of Health and Nutrition Biotechnology, Asia University, Taichung, Taiwan

**Keywords:** environmental tobacco smoke (ETS), Sirt-1, autophagy, Gerotarget

## Abstract

Recently, a survey by the Centers for Disease Control and Prevention (CDC) reported that nearly 90% of U.S. adult smokers began smoking at the age of 18. This demonstrates that the exposure to environmental tobacco smoke (ETS) of youngsters today is changing from passive smoking to active smoking (direct inhalation of tobacco). In the current study, an investigation of ETS exposure in young C57BL mice was conducted. After 6 weeks of ETS exposure, the Sirt-1 protein level was decreased and cardiac autophagy was increased in C57BL mice. Furthermore, the IGF2R cardiac hypertrophy signaling pathway was also triggered, although cardiac apoptosis and hypertrophy were not induced. Youngsters' desire to look more mature is one of the psychological factors that impacts smoking amongst young people. Our results suggest that though ETS exposure might cause cardiac autophagy amongst youngsters, the loss of the longevity Sirt-1 protein and the increase in IGF2R cardiac hypertrophy signaling could still promote heart diseases that are age-specific.

## INTRODUCTION

Environmental tobacco smoke (ETS) is defined as side-stream smoke, such as second-hand smoke, and main-stream smoke, such as cigarette smoke [1]. It was assumed that children were exposed to ETS, mainly second-hand smoke from their parents [2, 3]. However, recent research survey data from the Centers for Disease Control and Prevention (CDC) indicated that nearly 90% of U.S. adult smokers began smoking at the age of 18, suggesting that a large population of mainstream smokers start to develop from this age [4]. The Global Youth Tobacco Survey (GYTS) in 2010 which was similar to the United States survey, revealed that 24.6% of junior high students in Taiwan smoked cigarettes (Male = 30.6%, Female = 17.9%) [5]. Another study that targeted 50 schools in Europe, found that policies do not have a direct effect on the smoking behavior of adolescents aged 14-17 years in Europe [6].

In fact, the risk of smoking that affected cardiovascular health would increase for individuals who start smoking during their childhood [7]. Several studies have revealed the risks of cardiovascular disease (CVD) that are caused due to long-term ETS exposure. The GYTS discovered that deaths that were caused by smoking were twice the number of those who started to smoke before the age of eighteen [8, 9, 10]. In our previous study, we discovered that ETS exposure accelerated age-related cardiac disease and reduced the IGF-1 growth signaling in aging rat hearts [11, 12]. Furthermore, ETS exposure can disturb the oxidative processes in myocardial mitochondria and lead to cardiomyopathy [13]. The oxidative stress can also cause an expansion of defective mitochondria and induce mitochondrial fusion/fission or mitophagy [14, 15]. Under normal conditions, the role of autophagy in the heart is complex. However, evidence suggests that autophagy can be an adaptive mechanism under most conditions; a low level of autophagy eliminates damaged organelles and proteins to maintain cellular homeostasis [16]. However, crosstalk exists between autophagy and apoptosis in the development of heart disease [17].

Endogenous Sirt-1 I was involved in the cell death/survival process and the pathogenesis of CVD [18]. A heart failure model indicated that Sirt-1 could improve heart function by increasing AMP-activated protein kinase (AMPK) expression [19]. Furthermore, low Sirt-1 expression in aging contributed to the decrease of antioxidants and increase of proapoptotic molecules through oxidative stress pathways [20]. Furthermore, Sirt-1 directly deacetylated ATG5, ATG7, ATG8 and ATG12 proteins and led to autophagy in the heart [21]. Nevertheless, the roles of Sirt-1 in controlling autophagy and apoptosis in the heart were still multifaceted.

In the current study, we investigated the effects of 6 weeks of ETS exposure on young C57BL mice hearts. Interestingly, 6 weeks of ETS exposure did not cause cardiomyocyte apoptosis but led to reduction in Sirt-1 expression and increased autophagy in young C57BL mice. ETS-enhanced autophagy led to compensatory arousal of cardiomyocyte survival ability, but reduction of Sirt-1 expression accelerated the aging process in young C57BL mice.

## RESULTS

### Cardiomyocyte death analysis

**Figure 1:**
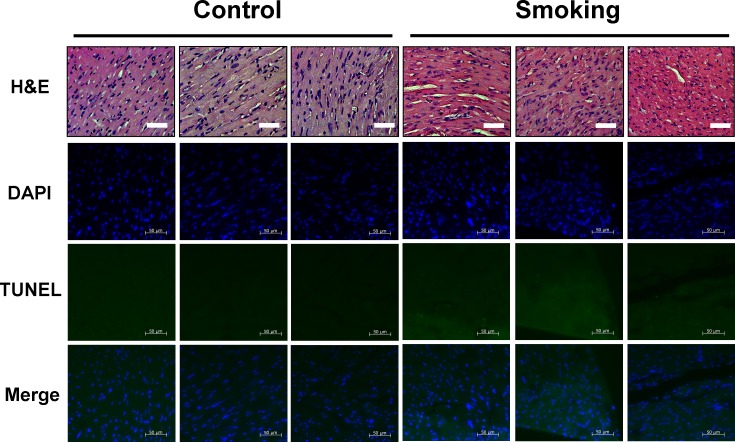
Biopsy of C57BL mice hearts **A.** After Hematoxylin and eosin (H&E) staining, cardiomyocytes nuclei are stained in blue, and other intracellular or extracellular proteins are stained in pink. All heart sections were obtained from the ventricular septum of each mouse (bar length = 50 μm). **B.** After DAPI and TUNEL staining, cardiomyocytes nuclei were stained in blue using DAPI on the heart biopsy slides, and specific DNA fragments in apoptotic cell nuclei were stained in green using TUNEL. (bar length = 50 μm).

After 6 weeks of ETS exposure, the morphology of cardiomyocytes of the ETS group did not change significantly when compared to those of the control group (Figure 1A). Furthermore, the cell apoptosis analysis (DAPI and TUNEL assay) also demonstrated that no apoptosis or cell death had occurred within 6 weeks in the ETS exposed group or control group mice (Figure 1B).

### Sirt-1 protein level and its deacetylase substrate protein analysis

**Figure 2:**
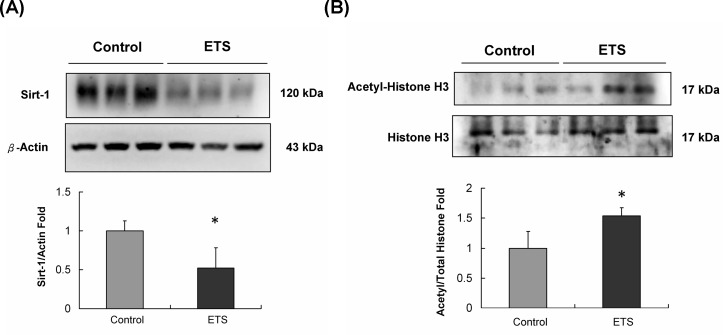
Sirt-1 protein level and its deacetylase substrate protein analysis **A.** Sirt-1 protein expression level was reduced in the ETS group mice hearts. **B.** The acetyl-histone H3 protein level was higher in the ETS group mice hearts, and this finding suggested that Sirt-1 lost its deacetylase potential after 6 weeks of ETS exposure in C57BL mice (*p < 0.05 compared with control group).

After 6 weeks, the Sirt-1 protein level in C57BL mice hearts was significantly reduced in the ETS exposure group when compared with the protein level in the control group (Figure 2A). The acetyl-histone H3 / histone H3 expression ratio in C57BL mice hearts was reduced in the ETS exposure group when compared with that in the control group after 6 weeks of ETS experiments (Figure 2B).

### IGF2R signaling pathway analysis

**Figure 3:**
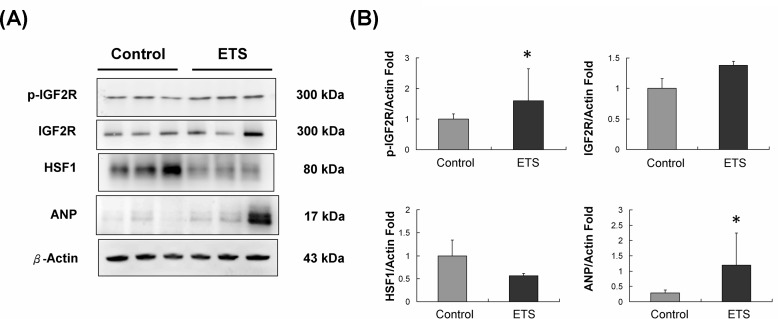
IGF2R signaling pathway analysis **A.** IGF2R and ANP protein levels were increased and HSF-1 protein levels were reduced after 6 weeks of ETS exposure in C57BL mice hearts. **B.** The quantification of protein expression in C57BL mice hearts (*p < 0.05 compared with control group).

After the 6-week experiments, the phosphorylated type IGF2R and downstream ANP protein level were slightly increased in the C57BL mice hearts of the ETS exposure group when compared with the hearts of the control group (Figure 3). Moreover, HSF1 protein expression was significantly reduced in the ETS exposure group C57BL mice hearts (Figure 3).

### IGFIR signaling pathway analysis

**Figure 4:**
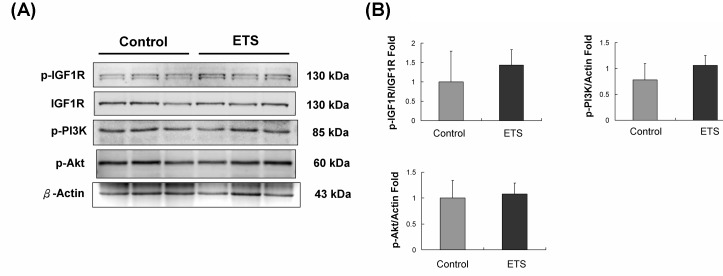
IGF1R signaling pathway analysis **A.** p-IGF1R, p-PI3K and p-Akt protein levels in C57BL mice hearts were not affected by 6 weeks of ETS exposure compared with those of the control group. **B.** The quantification of protein expression in C57BL mice hearts.

The IGF1R/PI3K/Akt pathway is a well-known cardiac survival pathway. After the 6-week experiments, p-IGF1R, p-PI3K and p-Akt protein expression levels did not increase in the control group C57BL mice hearts or in the ETS exposure group C57BL mice hearts (Figure 4).

### Autophagy expression analysis

**Figure 5:**
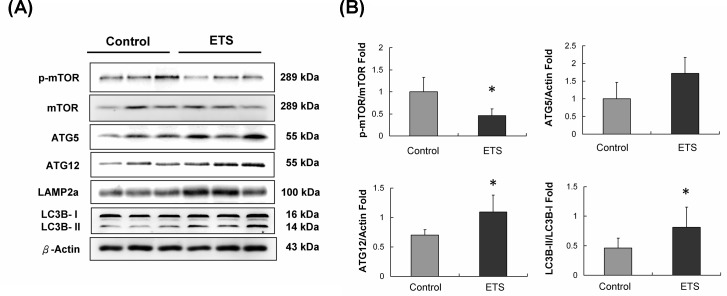
Autophagy initiated protein analysis **A.** ATG5, ATG12, LAMP2a and LC3B-II autophagic proteins were increased in ETS group mice hearts. **B.** The quantification of protein expression in C57BL mice hearts (*p < 0.05 compared with control group).

**Figure 6:**
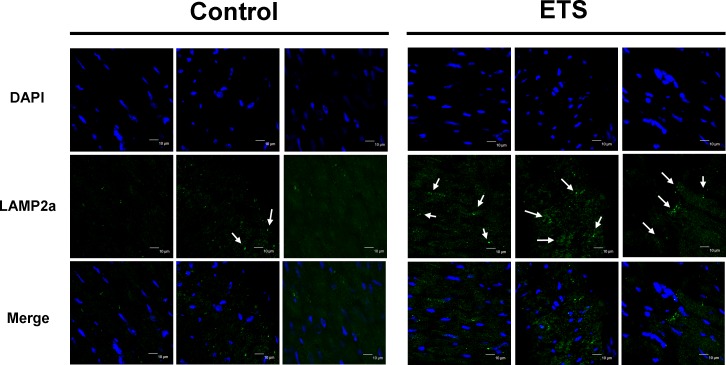
Autophagic vacuoles analysis Cell nuclei are stained in blue by DAPI assay, and lysosomal membrane protein, LAMP2a, is presented in green by an immunofluorescence assay. The formed autophagosomes are indicated by arrows (bar length = 10 μm).

After the 6-week experiments, the ratio of p-mTOR/mTOR was reduced in the ETS exposure group C57BL mice hearts (Figure 5). Furthermore, the ATG5 and ATG12 protein expression levels had increased in the ETS exposure group C57BL mice hearts. The LAMP2a protein levels and LC3B II/I ratio had also increased in the ETS exposure group C57BL mice hearts. In addition to this, fluorescent immunohistochemistry staining showed that LAMP2a was present in the autolysosomes of the heart tissue. The autolysosome number had increased in the 6-week ETS exposure group and was higher than those in the control group C57BL mice hearts (Figure 6).

### ETS treatment mice serum cultured cardiomyoblast H9c2 cells

**Figure 7:**
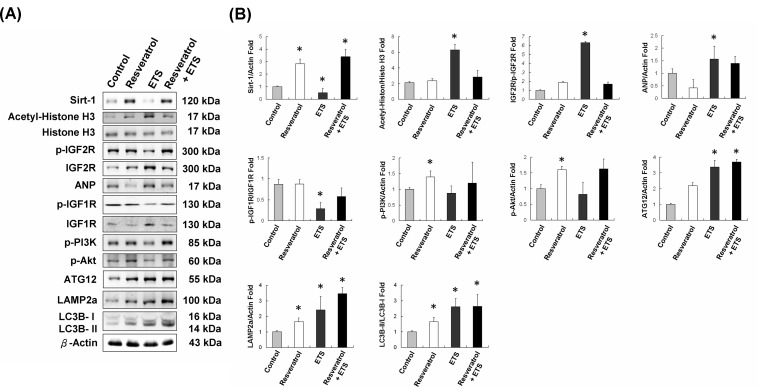
The proteins expressions of ETS mice serum cultured cardiomyoblast H9c2 cells **A.** The proteins levels of control, resveratrol, ETS and ETS combined with resveratrol mice serum cultured H9c2 cells **B.** The quantification of protein expression in mice serum cultured H9c2 cells (*p < 0.05 compared with control group).

Mice serum was collected from control and ETS group mice. After 10% mice serum in DMEM medium was cultured with H9c2 for 24h, the proteins expressions levels were analyzed and shown in Figure 7. The results showed that the IGF1R cardiac survival pathway and Sirt-1 expression activities were reduced in ETS group mice serum cultured H9c2 cells. Similar to the results presented in mice hearts, IGF2R and ANP expressions had increased in ETS group mice serum cultured H9c2 cells. Additionally, the Sirt-1 expression and activity had increased in ETS group mice serum combined with resveratrol (15μM) cultured H9c2 cells. But the autophagy in ETS group mice serum cultured H9c2 cells still could not be reversed by resveratrol (Figure 7).

## DISCUSSION

Previous studies have shown that exposure to ETS will reduce the cardiomyocyte survival rate and accelerate age-related cardiac disease in aging animals [11, 22]. However, cell death and cell apoptosis can not be detected in the ETS exposure group comprising C57BL mice hearts (Figure 1). Compensatory expression of IGF1R/PI3K/Akt cardiac survival signaling pathway components plays an important role in the protection of the hearts of animals when they suffer external environmental stress [23, 24, 25]. This finding should be the reason that approximately 6 weeks of ETS exposure did not affect the cardiomyocytes in mice hearts; the p-PI3K and p-Akt expression levels were almost the same in both the ETS exposure group and the control group (Figure 4).

Furthermore, Sirt-1 is a histone deacetylase (HDAC), which controls Akt signaling and is implicated in CVD and aging [26]. The acetyl-histone H3 is one of the substrate proteins for the deacetylase function of Sirt-1 [27]. Here, we analyzed the Sirt-1 protein level and acetyl-histone H3/histone H3 ratio between the control and ETS exposure groups in C57BL mice hearts. As the results showed, the Sirt-1 protein level and acetyl-histone H3/histone H3 ratio were both reduced in the ETS exposure group mice hearts (Figure 2). This finding suggests that 6 weeks of ETS exposure can reduce the Sirt-1 protein level and its deacetylase functions in C57BL mice hearts.

Although Sirt-1 is also considered to be a longevity factor through suppression of NF-κB -driven immune responses, the role of reduction of Sirt-1 in response to ETS exposure is still unclear and needs to be investigated further [28].

Another role of Sirt-1in autophagy is that it can directly deacetylate and activate ATG proteins [21]. In this study, the biomarkers of the cardiac autophagy signaling pathway, such as p-mTOR, ATG5 and ATG12, were increased in the ETS exposure group mice hearts (Figure 5). When autophagy starts, LC3B-1 will cleave and transform itself into LS3B-II during the autolysosomes processing. The LAMP2a expression and the LC3B II/I ratio confirm the formation of autolysosomes. Fluorescent immunohistochemistry staining of LAMP2a in heart slices reveal that the number of autolysosomes was significantly increased in the ETS exposed mice hearts (Figure 6). Recently, accumulating evidence revealed a relationship between cardiomyocyte autophagy and cardiac hypertrophy [29].

Our study showed that, the Sirt-1 degradation will reduce HSF1 in deacetylated DNA-binding state and increase IGF2R expression. Enhanced IGF2R expression will cause cardiac hypertrophy and cardiomyocytes apoptosis. [30]. This result suggested that the IGF2R signaling pathway played an important role in the development of cardiac hypertrophy. In this study, the expression of IGF2R signaling pathway components was increased in ETS exposed C57BL mice hearts, which caused magnified expression of atrial natriuretic peptide (ANP) (Figure 3). ANP was reported to be an endogenous biomarker of cardiac hypertrophy [31]. In addition to this, HSF1 acted as a repressor of IGF2R under normal conditions by binding to its heat shock element (HSE) in the IGF2R promoter region. It also protected cardiomyocytes by inhibiting IGF-IIR-induced apoptosis [30]. After the 6-week experiments, the HSF1 protein level was significantly decreased in ETS exposed C57BL mice hearts (Figure 3).

In this work, resveratrol as a Sirt-1 activator was used in ETS group mice serum cultured H9c2 cells. Although resveratrol can enhance the IGF1R cardiac survival pathway in H9c2, it can not reduce the autophagy in ETS group mice serum cultured H9c2 cells (Figure 7). This suggested that the ETS induced autophagy through an unknown mechanism and this might accelerate aging in hearts.

**Figure 8:**
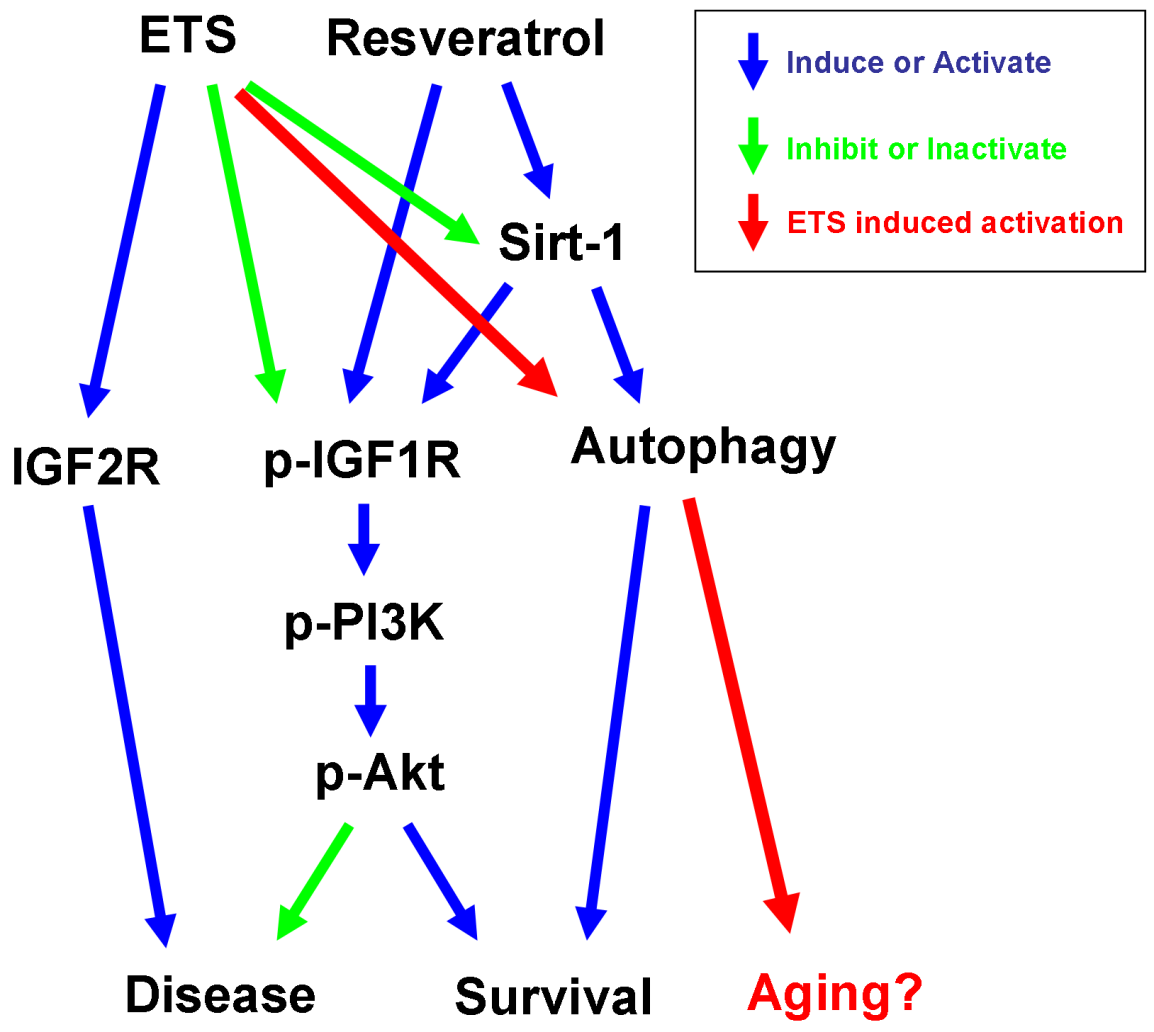
The ETS exposure will accelerate the aging and harm the heart A few autophagies that happen in healthy cardiomyocytes can help in removing the damaged organelles and maintain the physiological function of cells. Additionally, resveratrol can enhance autophagy during the aging process, but it can also provide an additional enhancement effect on IGF1R cardiac survival pathway in hearts. The autophagy induced by ETS exposure is entirely alien to resveratrol. The ETS exposure will increase the IGF2R more than the IGF1R signaling pathway, and further reduce the expression and activities of SIRT-. This proved that the ETS did not seem to affect the appearance; in fact, it could have accelerated the aging process and harmed the heart.

One of the psychological factors contributing to smoking amongst youth is the desire to look more mature, and this factor could explain the trend among youth to become active smokers rather than passive smokers [7]. In this regard, we found that ETS exposure in young animals would cause loss of expression of the longevity protein Sirt-1 and would accelerate the development of aging-induced CVD. In conclusion, our current findings show that ETS exposure can reduce the Sirt-1 level in hearts, enhance the activation of the cardiac hypertrophy-related IGF2R signaling pathway and enhance cardiac autophagy (Figure 8). Our results suggest that ETS exposure should be completely avoided rather than reduced in youth.

## MATERIALS AND METHODS

### Animals

The animal experimental protocol was approved by the Institutional Animal Care and Use Committee (IACUC) of China Medical University (No. 103-127-N). Twelve 6-week-old C57BL mice were purchased from the National Laboratory Animal Center (Taipei, Taiwan). The mice were divided into a control group (n = 6) and an ETS group (n = 6). All mice were fed with ordinary water and standard diets (Laboratory Rodent Diet 5001, LabDiet Co.). The mice in the ETS group were given ETS treatments twice daily (morning and afternoon) for 6 weeks. Each ETS treatment used 5 cigarette smoke of Long Life Gentle 7 brand cigarette purchased from Taiwan Tobacco & Liquor Corporation (Taipei, Taiwan). The ETS treatment procedure used a pump to collect 5 cigarettes' smoke into a chamber with 6 ETS exposure group mice; the mice were put in the chamber for 15 min ETS exposure and then fresh air was pumped in to replace the ETS. ETS group mice were then treated with ETS 2 times a day and this was repeated for 6 weeks. Control group mice were treated with the same procedure without ETS. After 6 weeks of experiments, the serum and heart tissue of mice were collected immediately and stored with liquid nitrogen for further analysis.

### Hematoxylin and eosin staining

After the above-described treatments were completed, the heart tissues from mice in each group were fixed in formalin for 7 days. Then, the heart tissues were dehydrated through graded alcohol treatments and embedded in paraffin wax. Later, 5 μm-thick paraffin sections were cut from these paraffin-embedded tissue blocks and transferred to gelatin coating slices. The tissue section slices were then deparaffinized by immersing them in xylene and then rehydrated. Next, all slices were stained with hematoxylin and eosin (H&E) and rinsed with water. Afterwards, slices were dehydrated by soaking them in graded alcohols and then transferring them twice to xylene. Finally, Photomicrographs were obtained using Zeiss Axiophot microscopes under 200× magnification.

### Fluorescent immunohistochemistry staining

After ETS treatments, the hearts from the mice in each group were immediately flash frozen in isopentane mixed with dry ice and kept at −70°C. Then, the tissues were embedded in an optimal cutting temperature compound (Tissue-Tek, SAKURA, Japan), and 5 μm cryostat sections were obtained. After the fixation, rehydration and blocking of the slides, the primary antibody LAMP2a (ab18528, Abcam, UK) was added for labeling the formed lysosome in heart sections. Then, the goat anti-rabbit IgG secondary antibody, Alexa Fluor^®^ 488 conjugate (A-11008, ThermoFisher, USA), was used to spot the fitted LAMP2a primary antibody. The cell nuclei were stained with DAPI as a final step before mounting the slice. The images were obtained using the Leica TCS SP2 confocal microscope detection system.

### Tissue protein extraction

Heart tissue protein extract samples from mice of each group were obtained by homogenizing the tissues using the PRO-PREP Protein Extraction Kit (iNtRON, Korea) lysis buffer. The homogenates were centrifuged at 13,000 x g for 30 min and kept at 4°C. The supernatants were collected and the protein concentration was determined using the Lowry protein assay. The protein concentration was adjusted to 40 mg/ml for further use.

### Cell culture

The cardiomyoblast cell line H9c2 was purchased from American Type Culture Collection (ATCC, CRL-1446, Rockville, MD, USA) and cultured in Dulbecco's modified Eagle's medium (DMEM, Sigma-Aldrich, MO, USA) with 2 mM glutamine and 10% fetal bovine serum in humidified air (5% CO_2_) at 37°C. The H9c2 cells were seeded in 6mm dishes at 2×10^5^ cells/dish with cultured medium for 4 h adherent; then they were changed to serum free medium for 12 h starvation. Next, the medium was added to 10% control group mice serum combined with 15 μM resveratrol. After 24 h treatments, the protein samples of these treated H9c2 cells were collected for western blotting analysis.

### Western blot assay

Heart tissue protein samples were analyzed by 12% SDS polyacrylamide gel electrophoresis (SDS-PAGE). Proteins were then transferred from the gel to Hybond-C membranes (GE Healthcare UK Ltd., Buckinghamshire, UK). After the protein transfer, the Hybond-C membranes were incubated in 3% bovine serum albumin (BSA) in tris-based buffer for 30 min. Primary antibodies, including p-IGF1R (ab-39398, Abcam, Cambridge, UK), IGF1R(ab39675, Abcam, UK), p-PI3K (sc-12929, Santa Cruz Biotechnology, USA), p-Akt (#9271, Cell Signaling, USA), p-IGF2R(ab138453, Abcam, Cambridge, UK), IGF2R (ab2733, Abcam, Cambridge, UK), HSF1(sc17757, Santa Cruz Biotechnology, USA), p-mTOR(#2974, Cell Signaling, USA), mTOR(#2983, Cell Signaling, USA), ATG5(#12994, Cell Signaling, USA), ATG12(#4180, Cell Signaling, USA), LAMP2a (ab18528, Abcam, UK), LC3B (#2775, Cell Signaling, USA),Sirt-1 (#9475, Cell Signaling, USA), Histone H3(#9715, Cell Signaling, USA), Acetyl-Hiatone H3(#4243, Cell Signaling, USA), and β-actin (SC-47778, Santa Cruz Biotechnology, USA), were added to the membranes for 1 hr at room temperature for protein identification. The membranes were then washed with TBS, and the secondary antibodies suggested for the primary antibodies were added to the membranes for 30 minutes. The secondary antibodies were then removed by TBS washing. Finally, horseradish peroxidase-labeled antibodies were added to the membranes, and images were obtained using the Fujifilm LAS-3000 (GE Healthcare UK Ltd.) analysis system.

### Statistical analysis

The results shown are the means ± SDs of three independent experiments. Statistical analysis was performed by one-way analysis of variance. For paired samples, Student's t test was applied.
